# The effect of implementing an aseptic practice bundle for anaesthetists to reduce postoperative infections, the Anaesthetists Be Cleaner (ABC) study: protocol for a stepped wedge, cluster randomised, multi-site trial

**DOI:** 10.1186/s13063-019-3402-8

**Published:** 2019-06-10

**Authors:** Alan F. Merry, Derryn A. Gargiulo, Ian Bissett, David Cumin, Kerry English, Christopher Frampton, Richard Hamblin, Jacqueline Hannam, Matthew Moore, Papaarangi Reid, Sally Roberts, Elsa Taylor, Simon J. Mitchell, the ABC Study Group, Ian Chapman, Tim Denison, Lara Hopley, Laura Jackson, Rachael Jones, Cornelius Kruger, Helen Lindsay, Andrew Love, Shay McGuinness, Paget Milsom, Jacob Munro, Guido Panduri, Rocco Pitto, Janie Sheridan, Navdeep Sidhu, Francois Stapelberg, Colin Sweetman, Jane Torrie, Stuart Walker, Christine Walsh, Tim Willcox, Siouxsie Wiles, Simon Young

**Affiliations:** 1 Department of Anaesthesiology, School of Medicine, University of Auckland, Private Bag 92019, Auckland, 1142 New Zealand; 2 Department of Anaesthesia, Auckland City Hospital, PO Box 92024, Auckland, 1142 New Zealand; 3 Department of Surgery, School of Medicine, University of Auckland, Private Bag 92019, Auckland, 1142 New Zealand; 4 Department of Surgery, Auckland City Hospital, Private Bag 92019, Auckland, 1142 New Zealand; 5 Department of Psychological Medicine, University of Otago, PO Box 4345, Christchurch, 8140 New Zealand; 6 Health Quality & Safety Commission, PO Box 25496, Wellington, 6146 New Zealand; 7 Department of Pharmacology and Clinical Pharmacology, University of Auckland, Private Bag 92019, Auckland, 1142 New Zealand; 8 Te Kupenga Hauora Māori, University of Auckland, Private Bag 92019, Auckland, 1142 New Zealand; 9 LabPLUS, Auckland City Hospital, PO Box 110031, Auckland, 1070 New Zealand; 10 Starship Children’s Health, Auckland District Health Board, PO Box 9389, Auckland, 1149 New Zealand

**Keywords:** Postoperative infection, Anaesthesia, Surgery, Patient safety, Prevention, Stepped wedge, Cluster randomised

## Abstract

**Background:**

Postoperative infection is a serious problem in New Zealand and internationally with considerable human and financial costs. Also, in New Zealand, certain factors that contribute to postoperative infection are more common in Māori and Pacific populations. To date, most efforts to reduce postoperative infection have focussed on surgical aspects of care and on antibiotic prophylaxis, but recent research shows that anaesthesia providers may also have an impact on infection transmission. These providers sometimes exhibit imperfect hand hygiene and frequently transfer the blood or saliva of their patients to their work environment. In addition, intravenous medications may become contaminated whilst being drawn up and administered to patients. Working with relevant practitioners and other experts, we have developed an evidence-informed bundle to improve key aseptic practices by anaesthetists with the aim of reducing postoperative infection. The key elements of the bundle are the filtering of compatible drugs, context-relevant hand hygiene practices and enhanced maintenance of clean work surfaces.

**Methods:**

We will seek support for implementation of the bundle from senior anaesthesia and hospital leadership and departmental “champions”. Anaesthetic teams and recovery room staff will be educated about the bundle and its potential benefits through presentations, written material and illustrative videos. We will implement the bundle in operating rooms where hip or knee arthroplasty or cardiac surgery procedures are undertaken in a five-site, stepped wedge, cluster randomised, quality improvement design. We will compare outcomes between approximately 5000 cases before and 5000 cases after implementation of our bundle. Outcome data will be collected from existing national and hospital databases. Our primary outcome will be days alive and out of hospital to 90 days, which is expected to reflect all serious postoperative infections. Our secondary outcome will be the rate of surgical site infection. Aseptic practice will be observed in sampled cases in each cluster before and after implementation of the bundle.

**Discussion:**

If effective, our bundle may offer a practical clinical intervention to reduce postoperative infection and its associated substantial human and financial costs.

**Trial registration:**

Australian New Zealand Clinical Trials Registry, ACTRN12618000407291. Registered on 21 March 2018.

**Electronic supplementary material:**

The online version of this article (10.1186/s13063-019-3402-8) contains supplementary material, which is available to authorized users.

## Background

Postoperative infection, which includes surgical site infection, pneumonia and sepsis, is a serious problem internationally with considerable human and financial costs [1, 2]. In New Zealand (NZ) alone the cost of postoperative infections exceeds NZ$136 million per year [1]. Surgical site infection occurs in up to 5% of “clean” operations [3]. Patients may face weeks with discharging wounds, time off work and re-admission to hospital [4]. In some cases, implanted artificial joints or heart valves may need to be removed and replaced. Haematogenous seeding of infection may lead to pneumonia. In the worst cases, sepsis may develop, with widespread inflammation, damage to organs and sometimes life-threatening “septic shock”.

In May 2017, the 70th World Health Assembly adopted a resolution on sepsis, urging member states “to reinforce existing strategies or develop new ones leading to strengthened infection prevention and control programmes, including… ...infection prevention practices in surgery” [5]. This followed a 2015 statement from the Lancet Infectious Diseases Commission which emphasised the global burden of sepsis and stressed global concerns over increasing pathogen resistance to antimicrobial therapies [6]. Worryingly, postoperative sepsis has increased in NZ from 7 per 1000 at-risk admissions between 2005 and 2009 to 11 per 1000 in 2013 [7]. The prevention of sepsis starts with the prevention of infections in general, notably after surgery. Thus, in NZ, reducing surgical site infection is a priority for the Health Quality & Safety Commission, the Accident Compensation Corporation, the Ministry of Health and the country’s district health boards, with national programmes implemented to this end. In 2016–2017, 28% of the Accident Compensation Corporation costs (NZ$15 million out of NZ$55 million) attributed to personal injury suffered during medical treatment were directed towards infections following surgery [8].

There is reason to believe that the burden of infective postoperative complications may not be distributed homogenously in NZ. For example, ethnic disparities in healthcare outcomes are substantial: health outcomes are, in general, worse in Māori and Pacific peoples than in their non-Māori/non-Pacific counterparts [9, 10]. Some factors identified as contributing to infection after surgery, including comorbidities such as obesity, diabetes [11] and skin infections, are more prevalent in Māori and Pacific populations [4, 12]. In addition, there could be differences in relevant aspects of their care both within hospital [13, 14] and outside hospital (e.g. access to care for wound reviews after going home from hospital). It therefore seems likely that Māori and Pacific peoples are at higher risk of postoperative infection.

Efforts to reduce postoperative infection have traditionally focussed on aspects of surgical technique and care, on hand hygiene, and on antibiotic prophylaxis. However, researchers in the United States [15–21] have recently demonstrated that anaesthesia providers also have a direct impact on bacterial transmission and infection rates in surgical patients. It is relevant that the work of anaesthetists may be very demanding. For example, anaesthetists administer a surprisingly high number of intravenously delivered medications (on average ten injections per patient [22] and frequently more), often under considerable time pressure. Similarly, techniques involved in securing patients’ airways to ensure adequate oxygenation after the administration of neuro-muscular blocking medications may be time-critical and technically difficult. In this setting, hand hygiene and the management of contaminated airway equipment may sometimes seem secondary to other more pressing requirements to keep patients alive. Anaesthetists’ hands frequently contact patients’ saliva whilst securing the airway, and blood during the insertion of lines into veins and arteries. Hand hygiene practices may be performed less than once per hour [16]. Anecdotal observation suggests that gloves are often seen as a substitute for hand hygiene and that, under pressure of workflow, anaesthetists often move from tasks that contaminate their gloves to adjusting their anaesthetic machines, handling syringes or undertaking various other activities. Similarly, contaminated laryngoscopes and other instruments may be inadvertently returned to clean surfaces rather than designated contaminated trays. One way or another, anaesthetists can rapidly and widely contaminate their work environment [17].

Cole et al. found that 12–16% of multi-use injection ports through which medications are administered contain bacteria within 6 h [23]. Crucially, Loftus et al. have demonstrated an association between contamination of these ports and postoperative mortality [20] and transmission of pathogenic bacteria from one patient on an operating list to another, including transmission via the hands of providers and environmental surfaces [24]. Recent data from our own group [25, 26] suggest that the methods by which anaesthetists typically draw up and adminster intravenous (IV) medications during surgery may be underestimated as a contributor to postoperative infection. In highly realistic simulated surgical cases using real medications and standard practices, our group cultured gram positive, gram negative and other micro-organisms associated with postoperative infections from 5 of 38 bags (13%) of collected injectate from IV medications [25]. Then, in a clinical setting at Auckland City Hospital, we asked anaesthetists to inject their IV bolus medications via a 0.2-μm filter unit inserted into the IV line of 300 patients undergoing surgery [26]. We isolated similar micro-organisms from 6% of these filters and from 2.4% of syringes retained for possible reuse during the anaesthetics. Thus, the injection of micro-organisms into patients may arise from deficiencies in aseptic techniques in the handling of IV medications as well as from contamination of IV injection ports. Loftus et al. have investigated the use of novel devices for hand sanitisation, device disinfection, IV catheter management and stopcock design, and demonstrated reduced bacterial contamination leading to a decrease in postoperative infection [16, 19, 21]. However, these approaches may not address failures in aseptic technique during the drawing up and injecting of IV medications. All sources of infection associated with these processes could be simultaneously and substantially addressed by injecting all IV boluses of medications through a commercially available 0.2-μm filter unit of the sort often used with epidural injections. Even if the injection port on one of these units becomes contaminated, it will be proximal to a filter that is fine enough to capture most micro-organisms [27]. We have shown that it is practicable for anaesthetists to inject most IV medications through these filters, with a rated difficulty of 3 out of 10 (10 being the most difficult) [26].

Unfortunately, it is not possible to manage the commonly used anaesthetic induction agent propofol in this way. Propofol is typically provided in lipid emulsion, composed of droplets with a mean (range) size of 0.19 (0.15–0.3) μm [28], and the manufacturers (Fresenius Kabi, AstraZeneca, and AFT Pharmaceuticals) recommend that the emulsion should not be injected through microbiological filters (manufacturers’ product information sheets). This is particularly problematic, because lipid emulsions provide a rich source of nutrients for bacterial growth. An association between propofol and postoperative infections began to be reported in the early 1990s [29–33]. In response, the then manufacturer provided an extensive educational programme for anaesthesia personnel and changed the product information to include explicit handling instructions. These instructions included using alcohol to decontaminate the neck of ampoules or rubber bungs of vials, drawing up the emulsion “aseptically”, maintaining single patient use and replacing infusion systems after 6 h [32]. Ethylenediaminetetraacetic acid (EDTA) was added to the formulation in 1996 [34]. EDTA does not prevent micro-organisms from contaminating the formulation, but it does retard their growth [35]. The product currently available in NZ does not contain any preservative and is presented in both vials and ampoules.

It seems, therefore, that a multi-faceted approach is called for to address the potential contribution of anaesthesia providers to postoperative infection. A strong precedent for the potential of a bundle of measures to reduce infection is to be found in the “Keystone Project”. This project involved strict adherence to a few simple, evidence-based practices in the insertion and subsequent management of central venous lines which substantially and sustainably reduced the median rate of catheter-related bloodstream infections per 1000 catheter days (from 2.7 pre-intervention to zero at 3 months) [36]. Widespread implementation of this bundle has been associated with reductions in mortality [37] and healthcare costs [38]. Impressive results were again obtained in a more recent study focussed on reducing catheter-associated bloodstream infections in patients with a bundle of care delivered by their intraoperative anaesthetic providers [39].

A key aspect of the “Keystone Project” was its use of the principles of implementation science [36]. Similar principles have been articulated in the recent World Health Organization publication “Guidelines on Core Components of Infection Prevention and Control Programmes” [40]. Drawing from these sources, guiding principles have been developed for this study (Table 1).

**Table 1:** The principles of implementation science guiding this study (adopted from Pronovost et al. [36])

1	The relevant practitioners should agree that the problem matters, and therefore that the response is warranted.
2	The evidence supporting or informing the requested practices should be convincing.
3	The tasks required should make sense, be possible to perform and preferably be easy to do.
4	Buy-in and support should be obtained at all levels, notably from practitioners and from senior clinical and managerial leadership: to this end as much engagement as possible should occur with all relevant participants at every stage of the implementation process.
5	Once the intervention has been agreed to, compliance should not be negotiable.

In line with principles 2, 3 and 4, we have worked collaboratively with anaesthetists, anaesthetic technicians, microbiologists and others from each of our study hospitals to develop an evidence-informed, multi-faceted practicable infection prevention bundle (see the following subsection) which combines a selection of key aseptic practices with the routine use of 0.2-μm filters for all IV bolus medications administered during anaesthesia, except propofol. We now aim to implement our bundle progressively in these institutions as a quality improvement project and to evaluate the impact of this implementation on the rate of postoperative infection.

### The development of the infection prevention bundle

Candidate elements for our infection prevention bundle were identified through a preliminary process of literature review and consultation with a group of relevant practitioners and other experts. A focus group meeting was then held with anaesthetists and anaesthetic technicians from each participating department. Rather than priming the participants with a suggested bundle, an open-ended invitation was given to make any suggestions on how to improve aseptic practices relevant to anaesthesia and to discuss any aspects relevant to any putative bundle. The importance of practicability and ease of implementation was emphasised by the facilitator, and no suggestions were disallowed. Notes were taken, and the preliminary bundle was modified by the investigators on the basis of reflection on this discussion. A second focus group was then held at which participants were presented with a draft of a proposed bundle. They were asked to comment on and discuss each aspect of this bundle. Notes were taken again. A penultimate draft of the bundle was then prepared, reflecting the published evidence, the discussions at both focus groups, the practicality and acceptability of each element of the bundle and the likely impact of these elements on postoperative infection.

### Primary hypothesis

We hypothesise that the implementation of a bundle that combines a selection of key aseptic practices with the routine use of 0.2-μm filters for all IV bolus medications administered during anaesthesia except propofol will reduce clinically relevant postoperative infections in a targeted group of high-risk patients. “Clinically relevant” in this context implies infections severe enough to require additional hospitalisation or death.

## Methods

### Ethics approval and informed consent

Approval has been obtained from the NZ Northern B Health and Disability Ethics Committee on the basis that this study will be an evidence-informed, quality improvement initiative and will not require informed consent from individual patients, because patient data will be used in an anonymous form, and the intervention is low risk. Instead we will obtain consent from participating anaesthetists, anaesthetic technicians and perfusionists, as recommended by Weijer et al. (2012) [41]. We will also seek site approval from each participating hospital, support from senior hospital leadership and agreement from participating departments to take part in the study.

### Design

Our study does not lend itself to the randomisation of individual cases. Instead, we will randomise clusters. To maintain separation of anaesthetists following usual practice from those who have adopted the aseptic practices in our bundle, we will cluster by departments. This implies a small number of large clusters. Quite substantial differences in practices and case mix may exist between departments, so intracluster correlation is likely to be quite high. Thus, we will use a real-world, multi-site, stepped wedge, cluster randomised quality improvement design (with five clusters) to compare outcomes when participants employ usual anaesthetic practices to outcomes when our bundle is adopted [42]. The stepped wedge design is ideal for progressive implementation of quality improvement initiatives such as the one in this study, and will also be more statistically efficient than alternative cluster designs because of our anticipated high intracluster correlation.

We will exclude data from our analysis for 1 week following transition from baseline to active phases of the study at each site to allow for the influence of progressive uptake of the bundle and the required changes in participating anaesthetists’ practice.

### Structure of the stepped wedge

All five sites will begin the study simultaneously on the first step, which will consist of normal care. Each step (in which one department adopts the bundle) will occur 6 months (180 days) after the previous one. Thus, at the end of the first 6 months the first site will adopt the bundle, whilst the other four sites continue with normal practice for another 6 months. The remainder of the sites will sequentially adopt the bundle in order; one every 6 months until 6 months after the start of the final (sixth) step. During the last 6 months all sites will be using the bundle.

The 6-month duration of the steps has been chosen because it allows enrolment of an adequate number of cases (see Statistics and sample size section), it ensures an adequate period within each new step for the bundle to become properly embedded and it allows sufficient intervals between steps for the rollout plan for each new step to be assiduously followed in an unhurried manner.

### Setting and participants

The study will be conducted in five departments (our clusters) in four large metropolitan hospitals in Auckland, NZ: Auckland City Hospital, Starship Children’s Hospital, Middlemore Hospital and North Shore Hospital (see Additional file 1: Table S1). The participants will be the clinical teams providing anaesthesia, perfusion and immediate postoperative care in the recovery unit for patients undergoing hip or knee arthroplasty or cardiac surgery in these hospitals during the duration of the study. These surgical subgroups have been chosen because these patients are subject to moderately high rates of postoperative infection, and the consequences of infection when it occurs are devastating — for example, implanted prostheses may need removal after arthroplasty, and sternal wounds may dehisce after cardiac surgery. Furthermore, well-developed systems for reporting surgical site infection to national databases are in place for these patients. We will be seeking approval for the study from senior hospital leadership and agreement in principle from participating departments to participate in the study, so it seems unlikely that an individual department will not adhere to the implementation of the bundle.

The projected case numbers per year have been taken from Surgical Site Infection Improvement Programme, national orthopaedic and cardiac surgical site infection reports (available from www.hqsc.govt.nz).

### Inclusion criteria

All hip or knee arthroplasty or cardiothoracic surgery (as defined by the Surgical Safety Infection Improvement Programme [43]) procedures will be carried out in the five clusters under general anaesthesia with or without regional anaesthesia, or under regional anaesthesia with sedation. Participant anaesthetists, anaesthetic technicians and perfusionists who consent to be part of the study will be included.

### Exclusion criteria

Patients receiving organs for heart and lung transplants will be excluded from the study because of their complexity and the use of immunosuppression in these cases. Likewise, patients donating organs for these purposes (i.e. patients who are classified as American Society of Anesthesiologists [ASA] 6) will be excluded. Participant anaesthetists, anaesthetic technicians and perfusionists who decline to be part of the study will be excluded.

### Withdrawal criteria

Participating anaesthetists will be expected to act in the best interests of their patients, and so will be free to withdraw individual cases or to omit any aspect of the bundle (for example, the use of the filter) if they believe this is warranted. Participants will be free to withdraw from the study. They will be asked to report any such decisions to the investigators.

### Intervention

The intervention will involve the implementation of the bundle as outlined below. It is recognised that some participating anaesthetists may already include some of these elements in their normal practice, so the bundle is *supplementary to usual practice* in that *all* of its elements are considered essential in the active phase of the study. In either phase of the study, if an anaesthetist’s standard practices include other steps to improve aseptic practice, these should be followed as usual for that clinician.

### The infection prevention bundle

**Fig. 1:**
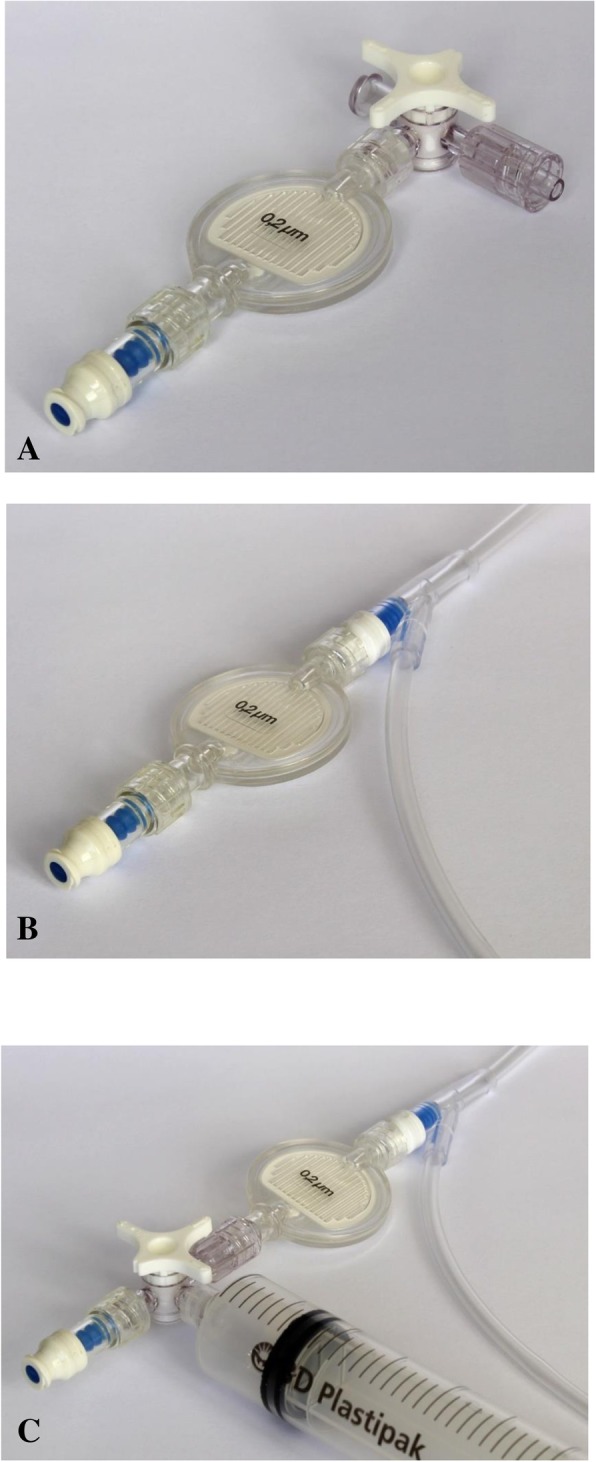
Three examples of filter configurations for the ABC study. **a** filter and injection port with a 3-way tap to be attached to the IV line; **b** filter and injection port attached to a side port on the IV line; **c**, as in **b**, with a 20-mL syringe filled with sterile sodium chloride 0.9% (for easy flushing) attached via a 3-way tap. Any practical approach that permits injection of medications through a filter is acceptable. Note: in these pictures the lines and filter are not primed with fluid. Fig. 1 is our own

The bundle consists of the following steps:

1. Wipe skin with alcohol (with or without chlorhexidine) and allow to dry before inserting any IV line.
2. Inject all IV bolus medications except propofol through a 0.2-μm filter incorporated into each patient’s IV line (see Fig. 1 for example configurations):

- Use aseptic technique when attaching the filter to the IV line and, unless it has been freshly opened from sterile packaging, wipe the IV line injection port to which the filter will be attached with alcohol (with or without chlorhexidine) for 15 s and allow to dry.
- If the filter is moved from one access point to another during the case, the new access point should first be wiped with alcohol (with or without chlorhexidine) for 15 s and allowed to dry.
- Use more than one filter if necessary or desired (e.g. for cardiac patients, one filter in the peripheral line, one on a central line port where bolus medications may be given, and a third onto the medication injection port on the bypass machine for the perfusionist to use when administering medications).
- Remove the filter(s) on discharge from the Post Anaesthesia Care Unit or on admission to the Intensive Care Unit.

3. Use a meticulous aseptic technique when drawing up or injecting propofol, and discard syringes, needles or the medication in the event of any suspected contamination:

- Note that the rubber bungs on propofol vials are not sterile even with the cap in place, so they should be wiped with alcohol (with or without chlorhexidine) for 15 s and allowed to dry before propofol is drawn up. If the medication is supplied in an ampoule, wipe the outside of the neck and surrounding part of the ampoule with alcohol (with or without chlorhexidine) before opening.
- Use a new needle or spike for each occasion.
- Cap the syringe with a syringe cap or capped needle.
- Administer as soon as possible and discard propofol after 1 h if not used.
- Do not reuse syringes or needles for propofol, even for the same patient.
- Flush the IV port with sterile sodium chloride 0.9% after propofol has been administered to ensure no residual propofol remains to support bacterial growth, using a meticulous aseptic technique to draw up the flush.

4. Perform hand hygiene:

- Before and after interacting with each new patient (i.e. on entering the operating room and on leaving a patient in the Post Anaesthesia Care Unit)
- Before and after any procedure creating risk of infection (e.g. IV insertion, airway manipulation, administering propofol, etc.).
- After blood and body fluid exposure (e.g. intubation, IV line insertion, etc.); remove gloves (if they have been worn) and, if practicable, perform hand hygiene before spreading contamination to the work station, computer key board and other surfaces.

5. Maintain clean working surfaces:

- Place used laryngoscopes, masks and other contaminated objects into a tray designated for this exclusive purpose; maintain strict separation of clean and contaminated areas — do not use this tray for clean instruments, swabs or other items even at the start of a procedure.
- Wipe the anaesthetic machine bench top and the circuit adjustable pressure valve with alcohol (with or without chlorhexidine) once the patient has settled into the maintenance phase of anaesthetic (i.e. after intubation of the trachea if this is done).

#### Notes

Specific notes regarding the infection prevention bundle are as follows:

- Propofol should not be injected through the filter.
- The filter has a dead space of 0.45 mL and the injection port has a dead space of 0.11 mL (= 0.56 mL in total); therefore, as with any IV setup, it is necessary to prime the filter with sterile sodium chloride 0.9% or sterile water for injection to eliminate air, and it is also necessary to ensure that medications are flushed through.
- Hand hygiene implies either hand washing with medicated soap and water or using alcohol-based hand rub; it is important for hands to dry properly.

Provided the medications are injected through a 0.2-μm filter, the study does not ask for hand hygiene in relation to the injection and drawing up of medications other than propofol (Fig. 1).

### Control (usual care)

For this study, usual care is defined as the practices usually used by each participating anaesthetist, notably in relation to asepsis. If a participating anaesthetist already uses some of the elements in the bundle, he or she should continue to do so.

### Both groups

It is expected that standard hospital policy will be followed in both groups with respect to:

- Operating room temperature control
- Surgical antimicrobial prophylaxis
- Single patient-use only for all medications and fluids
- Wiping the anaesthetic machine bench top and medication trolley top between each case
- The use of new or cleaned medication trays for each case.

It is expected that individual anaesthetists’ normal practice will be followed with respect to intraoperative oxygen concentrations and postoperative oxygen therapy.

### Implementation

The implementation will follow the principles of improvement science listed in Table 1.

### Development of educational and motivational material

The following resources have been developed:

1. An Anaesthetists Be Cleaner (ABC) study website (http://abc.auckland.ac.nz/) which includes the study synopsis, online resources for the study and up-to-date information on the details and progress of the study
2. A laminated two-sided single sheet outlining the bundle as above and including Fig. 1. This will also be placed in all participating operating rooms with the agreement of the participating departments, and on the study website
3. Sets of slides (including versions for online viewing) supported by handouts for presentations to participating anaesthetists, outlining the study, its rationale and its intervention
4. Demonstration videos contrasting ideal with imperfect practices in relation to the elements of the bundle. These have been made at the Simulation Centre for Patient Safety of the University of Auckland using highly realistic simulations of illustrative anaesthetic scenarios. The videos will be placed on the study website
5. A register of participants who have attended educational sessions on the study and watched the online videos.

### Enrolment of departments and departmental champions

Support in principle for this study was obtained from senior leadership in each of the participating hospitals. After ethics approval was obtained, the standard institutional site approval was sought from each hospital, which included obtaining the formal support of each department. After these formalities have been completed, the support of the chief executive officers and chief medical officers of each hospital, and permission to cite this for the purposes of the study, will be confirmed in writing. An email outlining the study and seeking support for it, with a copy of the protocol, will then be sent (through departmental administrators) to the staff of all participating departments, including all relevant surgeons and nurses. This will include an invitation for questions to be asked or concerns to be expressed. A study information sheet will be placed on the research notice board at each study centre, with a link to the study website.

One or more champions have already been appointed in each participating department. These champions will take part in and support all presentations and communications about the study.

### Randomisation of departments

The five departments will be sequentially allocated in a random order to the five possible dates of the bundle implementation, as shown in Table 2.

**Table 2:**
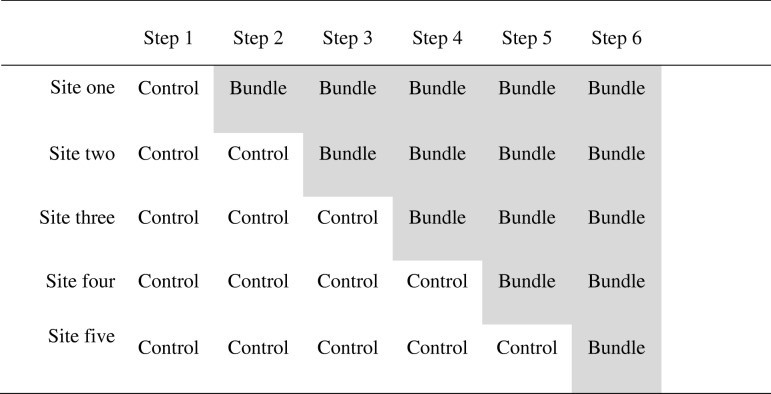
Implementation structure of the stepped wedge. *Shaded cells* indicate those steps and sites in which the intervention has been implemented

### Rollout of the bundle

The study will be rolled out sequentially across departments per the stepped wedge timetable outlined in Table 2. In the lead-up to each department’s rollout we will present the study to one or more departmental meetings (if necessary, further presentations will be made to technicians, perfusionists, nurses and surgeons, depending on interest, demand and availability to attend the primary presentations). At these presentations feedback will be sought on any ways to facilitate embedding the intervention, and questions will be invited. The aim will be to develop a sense of shared ownership and collaboration in implementing the bundle. The presenters will also inform participating anaesthetists and technicians that observational data pertaining to aseptic practices and adherence to the bundle once implemented will be collected. The anonymous nature of the data collected through observation will be emphasised.

Each department will be told of the date of the implementation of the bundle in the weeks leading up to that date. This is done to reduce the likelihood of a change in practice prior to the bundle implementation. During the weeks approaching the implementation date, communication with participating clinicians will be pursued to ensure that all relevant people are aware of the rollout. In particular, all participants will be sent one or more emails (through departmental administrators) reminding them of the planned rollout and inviting them to contact the investigators if they have any concerns or questions.

Posters promoting the study in general terms, and noting that observations may occur, will be placed in all participating departments and operating rooms several weeks prior to roll-out. These will have a section counting down the days to roll-out designed to alert all relevant people to the coming changes and to create some sense of anticipation and excitement. On the day of the rollout we will ensure that a poster with key elements of the bundle expressed in simple terms is placed in each relevant operating room and the post-anaesthesia care unit.

The study coordinator will liaise with the anaesthetic technicians in each department to ensure that the study consumables are available. For 4 weeks following the designated first day of the rollout, the study coordinator will be available in the operating rooms for at least some time each day to encourage the rollout, to deal with any difficulties and to answer any questions. Any practical difficulties will be recorded.

### Blinding, concealment and cross-contamination between study phases

Neither blinding nor concealment will be possible. The clusters have been chosen, in part, to minimise as far as possible any overlap of staff working in both control and active phases between sites. If this does occur, this will be noted and acknowledged as a limitation of the study.

### Outcomes and measures

Our interest in this study lies in demonstrating a reduction in clinically significant postoperative infections of any kind in our targeted high-risk patient group. Given our limited funding, we are not able to prospectively measure rates of infection ourselves, but we are able to access national and hospital databases of routinely collected relevant outcome data (see the following sections). In addition, we will collect observational data for each cluster both before and after implementation in order to assess changes in aseptic practices of anaesthesia team members over the period of the study.

#### Primary outcome

Our primary outcome variable will be days alive and out of hospital to 90 days (DAOH_90_). We have selected DAOH_90_ as our primary variable because:

1. It is sensitive to any postoperative complication (including, but not limited to postoperative infection) that is sufficiently serious to require prolongation of hospitalisation or re-admission into hospital or early death.
2. It is available from NZ’s national minimal dataset, and so can be readily collected for all patients.
3. It is more powerful, statistically, than a binary measure (such as infection vs no infection).

With our cluster randomised study design it will be reasonable to attribute any reduction in DAOH_90_ following implementation of the bundle to a reduction in clinically important postoperative infections of any type, and our analyses will make allowance for potential confounding factors unrelated to the study.

#### Secondary outcome

Our secondary (explanatory) outcome will be the rate of specified postoperative infections, as defined and collected by the national surgical site infection surveillance programme for patients undergoing hip or knee arthroplasty or cardiac surgery.

#### Process measures

The aseptic practices of the participating anaesthesia teams will be measured using a simple behaviourally anchored rating scale (BARS) (see Additional file 2). This has been developed for the study and will have elements addressing each item in our bundle. The overall score on the BARS for each case will provide our primary process measure. In addition, we will record participation by anaesthetists and technicians in relevant presentations, and in watching or reading online resources.

### Statistics and sample size

DAOH_90_ data have a bimodal, left-skewed distribution that does not lend itself to parametric analysis. Data from a previous study of 20,000 general surgery procedures indicate that most patients score highly, so comparing measures of central tendency between groups can exclude the patients who are most likely to realise improved outcomes. Although our preference would be to use permutation testing methods, our five-cluster, five-sequence design only allows for 120 permutations. We consider this too low a number for a precise estimation of the significance of the difference between control and intervention cases. We will thus use a single Wilcoxon-Mann-Whitney *U* (WMWU) rank sum test as our omnibus test to ascertain whether intervention patients have significantly higher DAOH_90_ than control patients. This WMWU test will also be sensitive to prevailing trends in patient outcomes, so we will further investigate the difference using quantile regression. Models will be fitted at quantiles 0.1, 0.25, 0.5 and 0.75, with DAOH_90_ as the outcome, and time, intervention and site as the predictors. We will characterise any differences in distribution between groups by reporting differences associated with the intervention term in each model, and their significance.

We have used simulations to estimate our statistical power. Datasets were synthesised using distributions generated from four of our sites (excluding Starship Children’s Hospital) with different step lengths. Starship Children’s Hospital data were synthesised on the basis of the distribution from Auckland City Hospital’s cardiac unit. Distributions for each site were tweaked by increasing or decreasing the relative likelihood of higher DAOH scores until the desired magnitude of difference appeared at quantile 0.25. We found that a step length of 180 days (i.e. 6 months) would give us 100% power to detect a 2 DAOH difference at quantile 0.25 between groups using the WMWU and 77.7% power to detect an intervention effect using quantile regression (both two-tailed α < 0.05), with just over 10,000 patients. The intracluster correlation coefficient was measured as 0.02. Cluster sizes in the simulation depended on the ratio of caseloads between sites (see Table 2). Our study statistician has estimated that we will recruit 5492 cases in each group and will require 8454 filter units.

We note that the study is not powered to show a difference in our secondary outcome, surgical site infection. Reducing surgical site infections from a base rate of about 1.4% (a reasonable estimate of the overall rate at present) to 0.8% would require approximately 11,000 patients (5500 in each group, one-tailed α = 0.05 and β > 0.8: estimated with MedCalc [MedCalc Software, Ostend, Belgium]). If we allow for cluster allocation by assuming a design effect of 2.0 (intracluster correlation coefficient = 0.02), the required number is likely to be double this. Given the likely proportion of Māori and Pacific patients, it is also not powered to investigate differences between these patients and other subgroups in the study, and any analysis of such differences will be exploratory and hypothesis-generating in nature.

An interim analysis will be performed after 12 months of data collection (i.e. at the end of Step 2, Table 2) to verify the safety of the intervention as indicated by an absence of serious adverse events related to the bundle. We will also verify the completeness of the study data. In addition, an independent monitor will be employed to verify that the study processes do protect the rights of the participants; the reported study data are accurate, complete and verifiable from source documents; and the conduct of the study is in compliance with approved protocol/amendment(s), with Good Clinical Practice and with the applicable regulatory requirements.

### Data collection

#### Outcome and patient-related data

Data will be requested from the Ministry of Health’s National Minimum Dataset (NMDS), the National Surgical Site Infection Improvement Surveillance Programme database and the databases of participating hospitals, and stored in a secure password protected directory within the Faculty of Medical and Health Sciences, University of Auckland. The data will be kept for ten years and then permanently deleted.

In NZ, each patient has a unique National Health Index (NHI) number, and each procedure has a specific International Classification of Diseases 10th Revision - Australian Modification (ICD-10-AM) code. The NHI will be used to link data from different sources and then replaced with a unique study identification number, in the interests of confidentiality.

For this study we will collect the following data: age, gender, ethnicity, weight, height, surgical procedure (including primary or re-operation status) and known comorbidities (with emphasis on those associated with risks of infection and failed wound-healing such as diabetes, severe renal disease, obesity and smoking) [11]. Uncontrolled significant differences in rates of these covariates between groups will be statistically accounted for using quantile regression.

Data for our primary outcome, DAOH_90_, will be requested from the NZ Ministry of Health and will include the date of discharge from hospital and the date of further re-admissions to hospital and will also indicate if the patient dies within the 90-day period.

Information on surgical site infection will be collected from the National Surgical Site Infection Improvement Surveillance Programme, which is limited to patients undergoing government-funded hip and knee arthroplasty and cardiac surgery. For these patients, a standard set of data is collected by trained hospital personnel (predominantly infection prevention and control nurses and perioperative nurses); the data are entered into the national database via an Internet portal. Strategies to ensure that all infections are identified include reviewing hospital microbiology records, having the ward teams alert the infection prevention and control team about suspected infections and reviewing the medical records of patients re-admitted to hospital within 90 days of the relevant procedures.

Patients who underwent their initial operation prior to the implementation of the bundle but are still in hospital during the implementation of the bundle will be included in the analysis of baseline data.

#### Adverse events and Data and Safety Monitoring Committee

Patients often experience adverse events related to surgery and anaesthesia, and recording and reviewing these events will not be practicable in this study. Any adverse event related or possibly related to the bundle that occurs before the patient leaves the post-anaesthesia area will be recorded on an Adverse Events Form. The primary investigators will be notified of any serious adverse events attributable to the study in writing within 24 h of becoming aware of such an event taking place.

Each adverse event related to the bundle will be classified by the primary investigators as:

- Non-filter-related adverse event
- Filter-related adverse event

Each filter-related adverse event will be assigned a severity classification as follows:

- *Mild*. Events cause awareness of signs or symptoms but are easily tolerated and are of minor irritant type, causing no loss of time from normal activities. Symptoms do not require therapy or a medical evaluation; signs and symptoms are transient.
- *Moderate*. Events introduce a low level of inconvenience or concern to the patient and may interfere with daily activities but are usually improved by simple therapeutic measures; moderate experiences may cause some interference with functioning.
- *Serious*. Events interrupt the patient’s normal daily activities and generally require systemic drug therapy or other treatment; they are usually incapacitating.

A Data and Safety Monitoring Committee will be formed and will be responsible for reviewing such serious adverse events as they arise.

#### Early termination

We do not expect any complications to arise from use of the bundle. In the unlikely event that any do, the investigators will liaise with the patient’s primary clinicians to manage any such complications. Then, taking into account the nature of the event, the certainty of association with the study intervention and the likelihood of recurrence, consideration will be given to terminating the study. Every effort will be made to avoid early termination for any reason other than safety.

In the event of early termination of the study, the Australian and New Zealand College of Anaesthetists, Becton, Dickinson and Company, the ethics committee and the trials registry will be notified.

#### Process data

At present, funding limits the extent to which we can evaluate current practices and changes to these practices after the implementation of the bundle. However, a small sample of cases will be observed at each site before and after implementation of the bundle to assess aseptic practices of the anaesthetic team using a BARS as described under the “Process measures” subsection. As indicated above, all participating staff (anaesthetists, anaesthetic technicians, surgeons and operating room nurses) will be informed of this aspect of the study at its outset, and relevant information will be included in the presentations to the departments and online. Individual consents will not be obtained.

Depending on availability, our observations will be done by people who are able to spend time in the operating room without necessarily attracting attention (e.g. medical students, nurses, anaesthetists or anaesthetic technicians) and who will be independent of the study in all other respects. Observations will be done on a pragmatic basis at times determined by the availability of these observers. Before starting the collection of baseline study data, preliminary observational data will be obtained to refine the BARS and establish its key properties, such as usability, reliability and interobserver repeatability. Within the time periods in which such staff are available for the study, we will randomly allocate the departments and operating rooms to be observed on any particular day, with stratification to ensure coverage of all the departments at each period of observation. As the study progresses, we will thus obtain information about both control and active phases of the study over time.

These observers will be trained in the use of the study BARS by one of the investigators. Training will also reinforce operating room etiquette and the need for the observations to be done discretely. Entire cases will be observed, producing one complete BARS form (and score) per case.

### Timeline

**Fig. 2:**
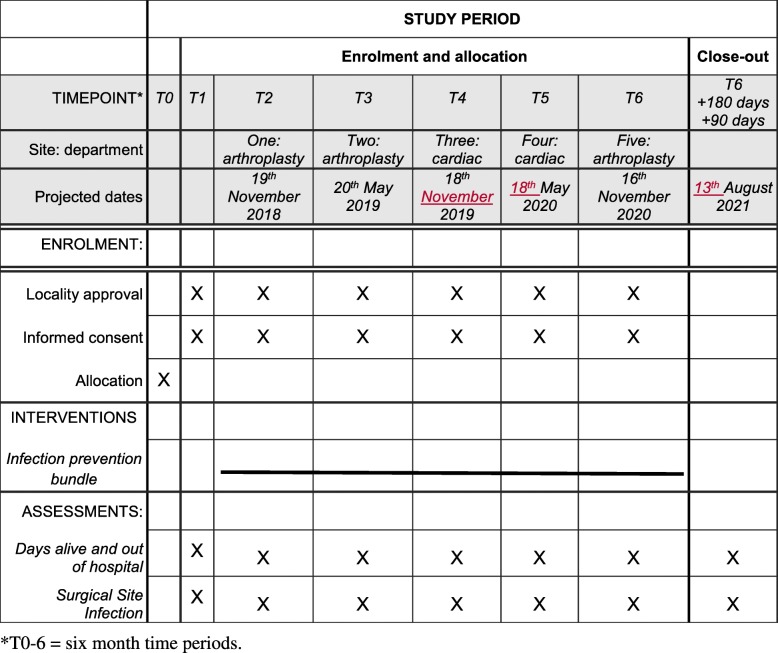
SPIRIT figure showing schedule of enrolment, interventions and assessments. Baseline data collection will be from 20 May 2018

The study is expected to run for 3 years from November 2018. We present a detailed schedule of enrolment, interventions and assessments as per the Standard Protocol Items: Recommendations for Interventional Trials (SPIRIT) guidelines (Fig. 2) [44]. The SPIRIT checklist is provided in Additional file 3.

## Discussion

Successful implementation of improvements in aseptic practices associated with anaesthesia has the potential to reduce postoperative infections. If this trial shows a positive change (i.e. an increase) in DAOH_90_ after implementation of our bundle, this will add to existing evidence suggesting that the practices of anaesthesia providers are a factor in the genesis of postoperative infections, and that improved outcomes can be achieved relatively easily. The investigators are well placed to liaise with relevant organisations to promote the subsequent adoption of the bundle throughout NZ. The potential benefits in relation to reducing patient harm and the costs associated with this are substantial, and the implications for Māori and Pacific patients may be particularly important.

Our bundle was designed to be simple and practicable. Some participants in our focus groups would have liked a more comprehensive bundle, aimed at perfect aseptic practice. In our view, more substantial changes in practice would be difficult to achieve, even in the context of a trial. Asking for too much may impede the readiness of participants to accept the bundle. It is also a strength that the evidence supporting our initiative is already known to many of our participants, in part because of previous studies undertaken on this topic in Auckland. In particular, the fact that the research into the potential role of the filters was both local and recent encourages us to believe that their potential value will be readily appreciated by participants.

One reason that the more general elements of the bundle have not already been more widely embraced may be the fact that infections that follow failures in aseptic practice manifest long after anaesthesia has finished. Thus, a postoperative infection is seldom, if ever, tracked back to the source, and there is no feedback to the anaesthetists about the consequences of such failures. In practice, it is quite difficult to achieve perfect aseptic practice during the dynamic and complex conduct of anaesthesia, and there are often more immediate threats to the patient. Greater motivation to maintain asepsis is required, and providing that motivation will be a key element of this initiative. Even with greater motivation, the required improvements in practice will need to be reasonably easy to implement, and as stated, we have strived to ensure that this applies.

What if our trial produces a negative result (i.e. no statistically significant change in DAOH_90_)? The question arises of how small a difference in DAOH_90_ actually matters? Might we miss a small difference that actually matters? The distribution of this variable is highly skewed and reflects the fact that most patients do not become infected and so will not experience any change in this outcome. We can expect the change, if it occurs, to be seen in the DAOH_90_ experienced by patients at lower centiles of the distribution. Thus, the difference may be more marked at the 25th percentile than at the 50th centile. With this in mind, our analysis will start by testing whether the distributions are significantly different or not (using a rank sum test tailored for a stepped wedge study that makes no assumptions about the nature of the distribution). If so, we will then provide numeric data on DAOH_90_ at the 10th, 25th, 50th, 75th and 90th centile and graphic representation of both distributions across the entire range to show where the effect lies. This approach avoids multiple testing and at the same time allows the maximum impact to be seen clearly without having to guess at the outset where it may lie (i.e. the 10th, the 25th or some other centile). We anticipate that this approach will mitigate the risk of missing clinically important differences between our groups.

We believe that DAOH_90_ may be a more powerful and more clinically relevant outcome measure for our study than the rate of infections per se. Nevertheless, we will utilize national databases to collect data on infections with a view to detecting any signal that will help attribute any differences in DAOH_90_ to differences in infection.

We believe the trial is well designed to test its primary hypothesis. The findings of this trial should add substantially to our understanding of postoperative infection. The trial is a direct response to the call by the World Health Assembly for new strategies “leading to strengthened infection prevention and control programmes, including… ...infection prevention practices in surgery” [5].

### Trial status

We are at Protocol version 1.3 (04 Sept 2018). Recruitment of participants began in October 2018. Implementation of the bundle commenced in November 2018, and data collection will end in May 2021.

## Additional files

Additional file 1: **Table S1.** Participating district health boards, hospitals and departments with the projected number of cases per year. (XLSX 10 kb)

Additional file 2: Tool for evaluating aseptic practices. (PDF 303 kb)

Additional file 3: SPIRIT 2013 checklist: recommended items to address in a clinical trial protocol and related documents. (DOC 121 kb)

## Abbreviations

ABC: Anaesthetists Be Cleaner
BARS: Behaviourally anchored rating scale
Cluster: Each of the five departments at the three district health boards
DAOH_90_: Days alive and out of hospital within the first 90 days after surgery
EDTA: Ethylenediaminetetraacetic acid
ICD: International Classification of Diseases
Bundle: Infection prevention bundle
IV: Intravenous
NZ: New Zealand
WMWU: Wilcoxon-Mann-Whitney *U*

## Authors’ information

AFM: Professor, Department of Anaesthesiology, University of Auckland and Consultant Anaesthetist, Auckland City Hospital, Auckland

DG: Research Fellow, Department of Anaesthesiology, University of Auckland

IB: Professor, Department of Surgery, University of Auckland and Surgeon, Auckland City Hospital, Auckland

DC: Senior Lecturer, Department of Anaesthesiology, University of Auckland

KE: Consultant Anaesthetist, Auckland City Hospital, Auckland

CF: Professor of Biostatistics, Department of Psychological Medicine, University of Otago, Christchurch

RH: Director, Health Quality and Evaluation, Health Quality & Safety Commission

JH: Lecturer, Department of Pharmacology and Clinical Pharmacology, University of Auckland

MM: Research Fellow, Department of Anaesthesiology, University of Auckland

PR: Tumuaki, Te Kupenga Hauora Māori, University of Auckland

SR: Clinical Microbiologist and Clinical Head of Microbiology, LabPlus, Auckland City Hospital

ET: Paediatric Anaesthetic Specialist, Starship Children’s Health, Auckland

SJM: Professor, Department of Anaesthesiology, University of Auckland and Consultant Anaesthetist, Auckland City Hospital, Auckland
