# Supplementary material for: The effect of implementing an aseptic practice bundle for anaesthetists to reduce postoperative infections, the Anaesthetists Be Cleaner (ABC) study: protocol for a stepped wedge, cluster randomised, multi-site trial
Source: Trials. 2019 Jun 10;20:342. doi: 10.1186/s13063-019-3402-8 (PMC6558820; doi:10.1186/s13063-019-3402-8)
Supplement: Supplementary file 2 — Tool for evaluating aseptic practices. (PDF 303 kb) [file 13063_2019_3402_MOESM2_ESM.pdf]

Date: ___________________ Procedure: ___________________

Case Number: ___________________ Rater: ___________________

- *The following elements should be scored based on the aseptic practice of the anaesthetic team (including technicians, medical students and nurses who are involved in the anaesthetic) rather than focusing on any one individual (i.e. the consultant anaesthetist).*
- *The examples of poor/ excellent technique should only be used to guide judgement of the team's aseptic practice, rather than be viewed as necessary criteria for a particular score.*

1. Insertion of intravenous lines in the operating room.

1 2 3 4 5

Poor **Excellent**

Examples of poor technique:
- Skin is not wiped with alcohol/chorhexidine before inserting IV line
- Skin is wiped with alcohol/chorhexidine but is then touched by hands with or without gloves

Examples of excellent technique:
- Skin is wiped with alcohol/chorhexidine before inserting IV line
- Skin is allowed to dry before inserting IV line

Comments:

2. Management of intravenous line components.

1 2 3 4 5

Poor **Excellent**

Examples of poor technique:
- When attaching, moving or reattaching segments of IV lines, ports, filters, Y-connectors, etc, the connecting elements are touched with fingers or are not wiped with alcohol/chlorhexidine and allowed to dry
- Port is left open after IV drug administration

Examples of excellent technique:
- When attaching, moving or reattaching segments of IV lines, ports, filters, Y-connectors, etc, the connecting elements are either freshly opened and therefore clean or are wiped for 15secs with alcohol/chlorhexidine and allowed to dry
- After IV drugs have been administered, the port is re-sealed to avoid contamination

Comments:

3. Aseptic technique when drawing up and injecting intravenous bolus medications (except propofol – see 4).

1 2 3 4 5

Poor **Excellent**

Examples of poor technique:
- Contamination occurs of IV access points
- IV access points are not wiped before use
- Alcohol/chlorhexidine wiping is for less than 15 seconds and or the wiped surfaces are not given time to dry
- Potentially contaminated needles or spikes are used to draw up the medication
- Medication syringes are not capped while kept for further use

Examples of excellent technique:
- All IV access points are either freshly opened and therefore clean or are wiped for 15secs with alcohol/chlorhexidine and allowed to dry
- Rubber bungs of vials or the necks of the ampoules are wiped for 15secs with alcohol/chlorhexidine before drawing up the contained medications
- The alcohol/chlorhexidine used above is allowed to dry before drawing up the medication
- Syringes for medications are capped while kept for further use
- Syringes, needles and medication are discarded in the event of any suspected contamination

Comments:

4. Aseptic technique when drawing up or injecting propofol.

1 2 3 4 5

Poor **Excellent**

Examples of poor technique:

- The vial rubber bung (or the neck of the ampoule) is wiped for less than 15secs with alcohol/chorhexidine
- The alcohol/chlorhexidine is not allowed to dry prior before drawing up propofol
- The same needle or spike is used on multiple occasions to draw up propofol
- The propofol syringe is not capped
- The same syringe or needle is used more than once to draw up propofol for the same patient
- The IV port is not flushed after propofol administration
- Propofol is retained for more than 30 minutes after being drawn up

Examples of excellent technique:

- The vial rubber bung (or the neck of the ampoule) is wiped for 15secs with alcohol/chorhexidine
- The alcohol/chlorhexidine is allowed to dry before drawing up propofol
- A new needle or spike is used on each occasion Propofol is drawn up, even for the same patient
- The syringe is capped
- The IV port is flushed after administration of propofol to avoid creating a reservoir of culture medium
- Syringes, needles and medication are discarded in the event of suspected contamination
- Propofol is administered within 30 minutes of being drawn up, or discarded

Comments:

5. Hand hygiene[a].

1 2 3 4 5

Poor **Excellent**

Examples of poor practice:

- Hand hygiene is not performed before and after interacting with each new patient, notably on entering the operating room
- Hand hygiene is not performed before and after any procedure which creates a risk of infection e.g. IV line insertion, airway manipulation, administering propofol
- Hand hygiene is not performed after blood and body fluid exposure e.g. intubation, IV line insertion.
- Gloves (if used) are not removed, and hand hygiene is not performed, before touching the work station, computer keyboard, mobile phone or other surfaces, and potential contamination behaviour occurs

Examples of excellent practice:

- Hand hygiene is performed before and after interacting with each new patient, notably on entering the operating room
- Hand hygiene is performed before and after any procedure which creates a risk of infection e.g. IV line insertion, airway manipulation, administering propofol
- Hand hygiene is performed after blood and body fluid exposure e.g. intubation, IV line insertion.
- Gloves (if used) are removed, and hand hygiene is performed, before touching the work station, computer keyboard, mobile phone or other surfaces

Comments:

6. Work surfaces.

1 2 3 4 5

Poor **Excellent**

Examples of poor practice:

- Separation of clean and contaminated areas is informal, unclear and poorly maintained
- Used laryngoscopes, masks and other contaminated objects are placed onto trays designated as clean or onto other clean work surfaces
- The anaesthetic machine bench top and the circuit pressure-relief valve is not wiped with alcohol/chlorhexidine once the patient has settled into the maintenance phase of the anaesthetic

Examples of excellent practice:

- Strict separation of clean and contaminated areas is explicit and maintained
- Used laryngoscopes, masks and other contaminated objects are placed only onto trays or other areas designated for contaminated items
- The anaesthetic machine bench top and the circuit pressure-relief valve is wiped with alcohol/chlorhexidine once the patient has settled into the maintenance phase of the anaesthetic

Comments:

a. Hand hygiene implies either washing hands with medicated soap and water or using alcohol-based hand rub to decontaminate them. Doing both would also be acceptable.
